# Genetic diversity and population structure in *Nothofagus pumilio*, a foundation species of Patagonian forests: defining priority conservation areas and management

**DOI:** 10.1038/s41598-020-76096-0

**Published:** 2020-11-06

**Authors:** M. Gabriela Mattera, Mario J. Pastorino, M. Victoria Lantschner, Paula Marchelli, Carolina Soliani

**Affiliations:** 1 Grupo de Genética Ecológica y Mejoramiento Forestal del Instituto de Investigaciones Forestales y Agropecuarias Bariloche (IFAB) INTA EEA Bariloche –CONICET, Modesta Victoria 4450, CP: 8400, S. C. Bariloche, Río Negro Argentina; 2 Grupo de Ecología de Poblaciones de Insectos del Instituto de Investigaciones Forestales y Agropecuarias Bariloche (IFAB) INTA EEA Bariloche –CONICET, Modesta Victoria 4450, CP: 8400, S. C. Bariloche, Río Negro, Argentina

**Keywords:** Forest ecology, Population genetics, Genetic variation, Conservation biology

## Abstract

Patagonian forests are the southernmost temperate forests in the world, and *Nothofagus pumilio* is one of their most ecologically important tree species (i.e., a foundation species). It presents great adaptability and a wide distribution range, making it a suitable model for predicting the performance of trees facing global climate change. *N. pumilio* forests are increasingly threatened by extreme climatic events and anthropogenic activities. This study aims to identify priority conservation areas and Genetic Zones (GZs) for *N. pumilio*, promoting the implementation of specific practices to ensure its management and long-term preservation. Thirty-five populations (965 trees) sampled across its distribution (more than 2200 km latitudinally) were genotyped with SSRs, and geographical patterns of genetic variation were identified using Bayesian approaches. The phylogeographic patterns of the species and geomorphological history of the region were also considered. Six priority conservation areas were identified, which hold high allelic richness and/or exclusive allelic variants. Eighteen GZs were delineated based on the genetic structure of this species, and maps showing their distributional range were drawn up. Overall, this study defines management units based on genetic data for *N. pumilio* for the first time, which will facilitate the establishment of sustainable practices and highlight priorities for investment of conservation funding.

## Introduction

Patagonian forests represent the most southern temperate forests in the world, and were isolated from other continental forests approximately 40–2.5 million years before present^1^. The biological value of these forests lies partly in their high level of endemism and frequency of taxonomically isolated genera, despite low species richness in comparison with other South American forests^1^. Together with some conifer species, southern beeches of the Nothofagus genus constitute the main components of Andean Forests, which are recognized as providing a wide range of ecological, economic, and social benefits and services. Therefore, the management of these native forests is required in order to maintain the ecological dynamics of this unique ecosystem. Several studies have documented the role of climatic variability during the Quaternary in shaping the current distribution of Patagonian forests^2–4^. In addition, extreme climatic events have recently been suggested as one of the causal agents of current forest decline in Northern Patagonia^5^, e.g. severe droughts occurring after a wet period triggered *N. pumilio* decline^6^. In this regard, an increase in mean maximum summer temperature, overall warming, and a marked decrease in annual precipitation were detected in this region throughout the twentieth century^7,8^, threatening the persistence of these forests. Besides, anthropogenic and natural causes such as logging and more frequent fires^9,10^ also endanger these forests.

*Nothofagus pumilio*, commonly called lenga, is one of the most important species of Patagonian forests, since it has the largest distribution area of all native trees in the region, covering about 1.636 million hectares and occupying a range of about 20° latitude (from 36°S to 55°S) in Argentina. This species has a similar distributional range to the west of the Andes mountain range, in Chile^11^. Moreover, it occurs in heterogeneous environments along altitudinal, latitudinal, and longitudinal gradients that imply diverse and contrasting thermal, photoperiodic, and precipitation conditions, respectively. *N. pumilio* shows great adaptation or plasticity to extreme and contrasting environments, since it grows close to the timberline (cold-tolerant species) and thrives in a wide range of precipitation levels (from 3000 to 300 mm average annual precipitation)^12^. In addition, several phenotypic variations have been described for this species across environmental clines; e.g., bud phenology variation and seedling growth associated with altitude^13,14^, and wood density related to soil moisture gradients^15^. Since *N. pumilio* exhibits great adaptability, it serves as a suitable model for prediction of the performance and fitness of trees to cope with global climate change in the southern hemisphere. Consequently, a research is currently being undertaken to determine whether local adaptation of this Southern beech evolved in a convergent way with respect to European tree species that are also distributed along broad environmental gradients (bi-national project, grant number 410759149, German Research Foundation-DFG). Natural hybridization between *N. pumilio* and *N. antarctica* has been observed in sympatric populations^16,17^ and it has been suggested that the latter acts predominantly as pollen receptor^18^. The introgression of the chloroplast genome from *N. pumilio* to *N. antarctica* would have occurred as a consequence of repeated backcrosses between hybrids and parents, and the former is clearly distinguished at the nuclear level^19^.

### The importance of defining operational genetic management units and priority conservation areas in *N. pumilio* forests

Government directives regulating the criteria and indicators for defining management units, such as Conservation Units (CU), became important worldwide during the last decades of the twentieth century. Appropriate management of native resources depends on understanding the nature and distribution of genetic diversity among and within species, and highlighting how this genetic variation is structured across their distribution range. Since most native forests are widely distributed and composed of hundreds of natural populations, management of their genetic resources cannot be based on single-population units. Thus, the establishment of operational genetic management units (OGMUs) is necessary for implementation of specific practices arising from conservation or restoration programs. In Argentina, OGMUs were delineated in three native species based on genetic criteria: *Austrocedrus chilensis*^20,21^, *Nothofagus nervosa* and *Nothofagus obliqua*^22,23^. According to the National Law N° 26331 on Minimum Requirements for Environmental Protection of Native Forests, most of the Argentinean *N. pumilio* forests (up to 97%) were included in areas of high (38%) or moderate (59.1%) conservation value^24^. Therefore, forest management is highly encouraged for this species, particularly the identification of priority conservation areas, in order to ensure compliance with this law and their long-term preservation. The identification of Genetic Zones (GZs) is one alternative of OGMU delineation. GZs are genetically homogeneous regions within which seeds can be transferred with low risk of causing change in the original genetic structure^25,26^. A requirement for the identification of GZs is the characterization of the population structure of a species, resulting from genetic signatures of evolutionary history, which can be assessed by neutral molecular markers. A second, well-known alternative for OGMU delineation is the definition of Provenance Regions (PRs), which aims to ensure the safe transference of propagation material without risk of maladaptation since it is based on variation in adaptive characters^27^. In addition, assessment of species’ genetic diversity with molecular markers could facilitate the identification of priority conservation areas^28,29^. Several authors have suggested that areas containing high allelic richness and/or exclusive allelic variants are meaningful for conservation purposes^30–32^, particularly because they play a key role as genetic diversity reservoirs or sources of locally-adapted genotypes.

The aim of this work is to identify priority conservation areas in *N. pumilio* forests. Genetically homogeneous regions (i.e., GZs) were first delineated by assessing the neutral variation of the target species across its natural distribution range in Argentina. Based on this, geographical patterns of *N. pumilio* genetic variation were recognized. In addition, areas holding high allelic richness and/or exclusive allelic variants were identified. Previous information from *N. pumilio* phylogeographic patterns^33^ was considered and new information from SSR markers was added.

## Results and discussion

### Intra-species genetic diversity

**Figure 1:**
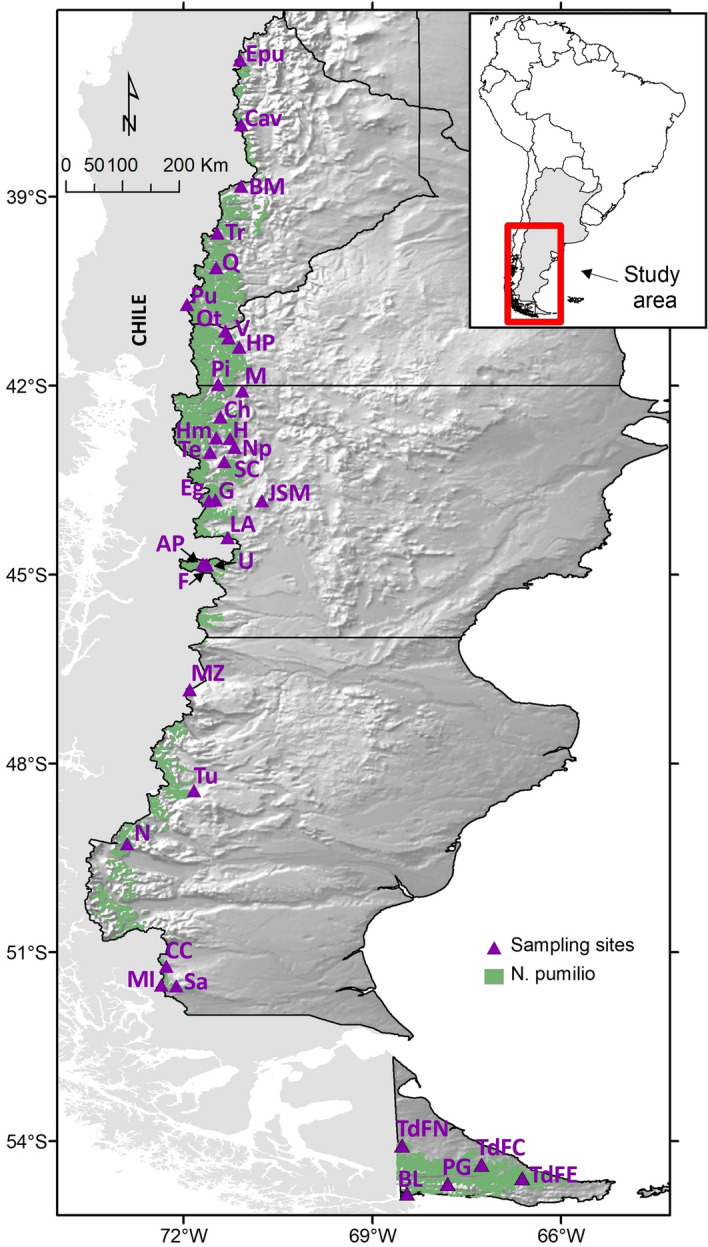
Current distribution range of *Nothofagus pumilio* in Argentina and location of the sampled populations used in this study. Population IDs correspond to those presented in Table 1. The map was created using ArcGIS 9.2 (ESRI, https://www.esri.com).

The characterization of intraspecific genetic diversity patterns is crucial for development of sustainable forest management prescriptions^34^, due to its usefulness in determining the persistence and adaptive potential of a species. Therefore, indicators of genetic diversity should be considered when developing criteria and guidelines for forest management^35–37^. A set of genetic parameters was used here in order to characterize the *N. pumilio* populations (Table 1, Fig. 1).

**Table 1:** Genetic diversity parameters estimated for each *N. pumilio* population (35).

Population name	ID	Lat (S)	Long (W)	N	*N*_*a*_	*N*_*e*_	*H*_*E null*_	*H*_*E Nei*_	W-test	*F*_*IS*_′	*F*_*IS*_′′
Epulauquen*	E	36° 50*′*	71° 06*′*	32	7.00 ± 1.48	3.94 ± 0.86	0.712	0.725	ab	0.0781	0.0000
Caviahue*	Cav	37° 51*′*	71° 05*′*	26	7.43 ± 1.66	3.98 ± 0.89	0.672	0.712	ab	–	–
Batea Mahuida	BM	38° 50’	71° 05’	25	6.43 ± 1.02	3.20 ± 0.65	0.633	0.661	abc	–	–
Tromen*	Tr	39° 35*′*	71° 28´	34	8.57 ± 1.85	5.00 ± 1.22	0.684	0.757	a	0.1044	0.0000
Quilanlahue*	Q	40° 08´	71° 29´	29	7.43 ± 1.84	4.11 ± 1.20	0.622	0.707	abc	–	–
Paso Puyehue	Pu	40° 42’	71° 56’	22	6.43 ± 1.41	3.48 ± 0.91	0.637	0.613	abc	–	–
Cerro Otto	Ot	41° 08´	71° 20´	20	5.86 ± 1.22	3.79 ± 1.02	0.651	0.691	abc	–	–
Valle del Chalhuaco*	V	41° 15´	71° 17´	29	6.00 ± 1.29	4.25 ± 1.16	0.644	0.673	abc	0.1003	0.0000
Ea. Herodina Parada	HP	41° 23´	71° 7´	24	4.14 ± 1.39	2.38 ± 0.76	0.386	0.398	c	–	–
Cerro Piltriquitrón	Pi	41° 58´	71° 27´	22	5.86 ± 1.35	3.13 ± 0.99	0.580	0.573	bc	–	–
Mina de Indios	M	42° 05´	71° 04´	20	5.57 ± 1.39	3.13 ± 0.65	0.711	0.649	abc	0.0212	0.0000
Cholila	Ch	42° 30´	71° 25´	18	5.00 ± 1.11	2.70 ± 0.47	0.567	0.594	abc	0.1470	0.0001
Huemules*	Hm	42° 50´	71° 29´	30	7.43 ± 1.66	3.90 ± 1.05	0.664	0.670	abc	–	–
La Hoya*	H	42° 50´	71° 16´	31	6.00 ± 1.15	3.34 ± 0.58	0.673	0.676	abc	0.1447	0.0000
Cerro Nahuelpan*	Np	42° 59´	71° 11´	29	6.86 ± 1.16	4.56 ± 1.17	0.755	0.702	abc	0.2949	0.1461
Trevelin*	Te	43° 04´	71° 35´	30	6.86 ± 1.14	3.75 ± 0.52	0.738	0.721	abc	–	–
Sierra Colorada	SC	43° 12´	71° 20´	30	5.57 ± 1.25	3.70 ± 0.82	0.682	0.657	abc	–	–
Lago Guacho*	G	43° 49´	71° 30´	30	7.00 ± 1.36	3.31 ± 0.67	0.615	0.611	abc	–	–
Lago Engaño*	Eg	43° 50´	71° 35´	43	6.57 ± 1.65	3.86 ± 1.18	0.607	0.627	abc	0.1199	0.0020
José de San Martín*	JSM	43° 50´	70° 45´	32	5.71 ± 1.21	2.87 ± 0.61	0.599	0.617	abc	–	–
Lago Azul	LA	44° 25´	71° 18´	20	4.57 ± 0.75	3.00 ± 0.58	0.603	0.613	abc	–	–
Arroyo Perdido*	AP	44° 50´	71° 42´	32	5.57 ± 1.00	3.04 ± 0.76	0.590	0.608	abc	0.0730	0.0000
Lago Fontana*	F	44° 50´	71° 38´	32	6.43 ± 1.56	4.15 ± 1.18	0.650	0.649	abc	0.1416	0.0001
Río Unión*	U	44° 51´	71° 39*′*	30	5.86 ± 1.20	3.67 ± 0.95	0.644	0.689	abc	0.0534	0.0001
Monte Zeballos	MZ	46° 50’	71° 54’	20	6.57 ± 1.17	3.68 ± 0.83	0.648	0.662	abc	–	–
Ea. Tucu Tucu	Tu	48° 26’	71° 50’	20	5.71 ± 1.17	3.51 ± 0.82	0.602	0.591	abc	–	–
El Chaltén	N	49° 17’	72° 54’	19	6.43 ± 1.65	3.45 ± 0.95	0.615	0.625	abc	–	–
Cancha Carrera*	CC	51° 13*′*	72° 16*′*	29	6.43 ± 1.43	3.61 ± 0.86	0.652	0.688	abc	–	–
Mina I*	MI	51° 31*′*	72° 21*′*	30	6.71 ± 1.57	3.89 ± 0.96	0.641	0.684	abc	–	–
Punta Gruesa	Sa	51° 32*′*	72° 07*′*	26	5.43 ± 1.09	3.23 ± 0.58	0.637	0.637	abc	–	–
Tierra del Fuego Norte*	TdFN	54° 05*′*	68° 32*′*	30	7.00 ± 1.83	3.69 ± 1.00	0.648	0.677	abc	–	–
Tierra del Fuego Centro*	TdFC	54° 22*′*	67° 16*′*	29	6.71 ± 1.04	3.76 ± 0.64	0.692	0.678	abc	–	–
Tierra del Fuego Este*	TdFE	54° 35*′*	66° 37*′*	32	6.29 ± 1.19	3.62 ± 0.75	0.657	0.686	abc	0.1075	0.0000
Paso Garibaldi	PG	54° 41*′*	67° 48*′*	29	5.57 ± 1.32	2.78 ± 0.57	0.566	0.581	bc	–	–
Bahía Lapataia	BL	54° 50*′*	68° 27*′*	30	4.57 ± 1.02	2.39 ± 0.58	0.519	0.537	c	–	–

*Lat* (*S*) south latitude, *Long* (*W*) west longitude, *N* sample size, *Na* number of different alleles, *Ne* effective number of alleles, *H*_*E null*_ corrected *HE* (expected heterozygosity) considering null alleles, *H*_*E Nei*_ Nei’s unbiased heterozygosity obtained through bootstrap re-sampling procedure, *W-test* Wilcoxon sign-rank test, *FIS*′ inbreeding coefficient considering null alleles using Bayesian methods (considering the best model), *FIS*ʺ inbreeding coefficient considering null alleles using Maximum likelihood. *These populations were also included in Soliani et al.^19^.

Significant differences in unbiased heterozygosity were detected among populations using Wilcoxon’s test (*p* < 0.05). In particular, *H*_*E Nei*_ for Tr population (0.757) was significantly higher than the *H*_*E Nei*_ obtained for PG (0.581), Pi (0.573), BL (0.537), and HP (0.398) populations. In addition, both BL and HP exhibited the lowest heterozygosity values, suggesting that they hold lower genetic diversity than the other populations analyzed.

Inbreeding and bottlenecks are demographic events that shape population genetic variation, specifically, reducing allelic variants. Signs of inbreeding were detected in 12 populations (Table 1), but only Np showed a significant inbreeding coefficient after standard errors of the estimates were calculated by jackknifing across loci (*F*_*IS*_*”* = 0.1461, Z > 1.96). Since this population is isolated from the main forest masses (western side of the species’ distribution) and located in an area that was covered by an ice field at the Last Glacial Maximum (LGM), we hypothesize that few individuals contributed to the recolonization process. In addition, a population bottleneck was identified in Sierra Colorada (SC), since it showed significant heterozygosis excess under both Infinite Allele Model -IAM and Two Phase Model -TPM mutation models (Wilcoxon’s test, *p* < 0.05).

The differentiation coefficient for *N. pumilio* was estimated from the *F*_*ST*_ index with and without implementing the ‘exclusion of null alleles’ (ENA) method (0.077 [0.050–0.113] and 0.078 [0.053–0.115], respectively). The indexes did not differ substantially, indicating that null alleles did not strongly bias the *F*_*ST*_ value. At the same time, the standardized Hedrick’s *G′*_*STH*_ (calculated based on the *G*_*ST*_ coefficient = 0.075) was 0.201. These differentiation coefficients (*F*_*ST*_ and *G*_*ST*_) were similar to those previously reported in other *Nothofagus* species such as *N. nervosa* (0.061) and *N. obliqua* (0.049)^22^.

An isolation-by-distance (IBD) pattern was not supported by the Mantel test and the Spatial Genetic Structure (SGS) test (see Supplementary Fig. S1), in accordance with a previous study that involved 20 *N. pumilio* populations^19^. Based on these results, an estimation of population structure was carried out, implementing spatial clustering methods as recommended by Perez et al.^38^. In contrast, the genetic and geographic distances do correlate with each other when using maternally inherited cpDNA markers^33^. In this regard, it was suggested that the pollen dispersal capacity of *N. pumilio* greatly exceeds that of seed dispersal (75–100 times greater)^39^, as in other wind-pollinated trees^40,41^, which could partly explain the differences found between cpDNA and SSR results from IBD analyses.

### Cluster definition and genetic zone delineation

**Figure 2:**
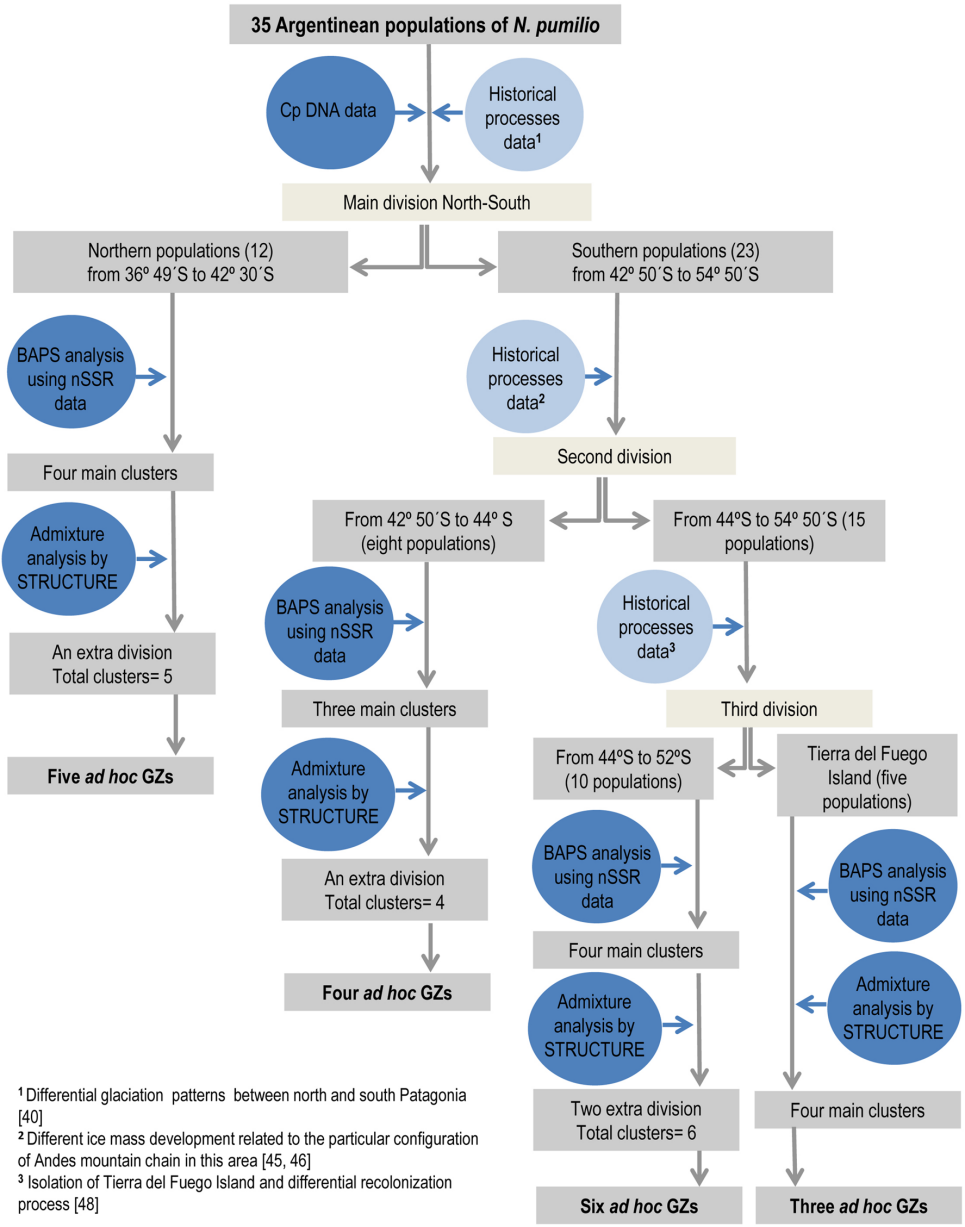
Flow chart summarizing the decision-making process involved when delineating *N. pumilio* Genetic Zones.

Our results showed that a main division between northern and southern chloroplast lineages took place between Cholila (42° 30*′*S) and Huemules (42° 50*′*S) in *N. pumilio* forests, since all individuals analyzed from these two sites exhibited either northern or southern haplotypes, respectively. The evolutionary significance of this lineage segregation prompted us to consider these findings as the first division criterion for exploration of the genetic structure of Argentinean *N. pumilio* forests located to the north and south of these boundaries (Fig. 2).

**Figure 3:**
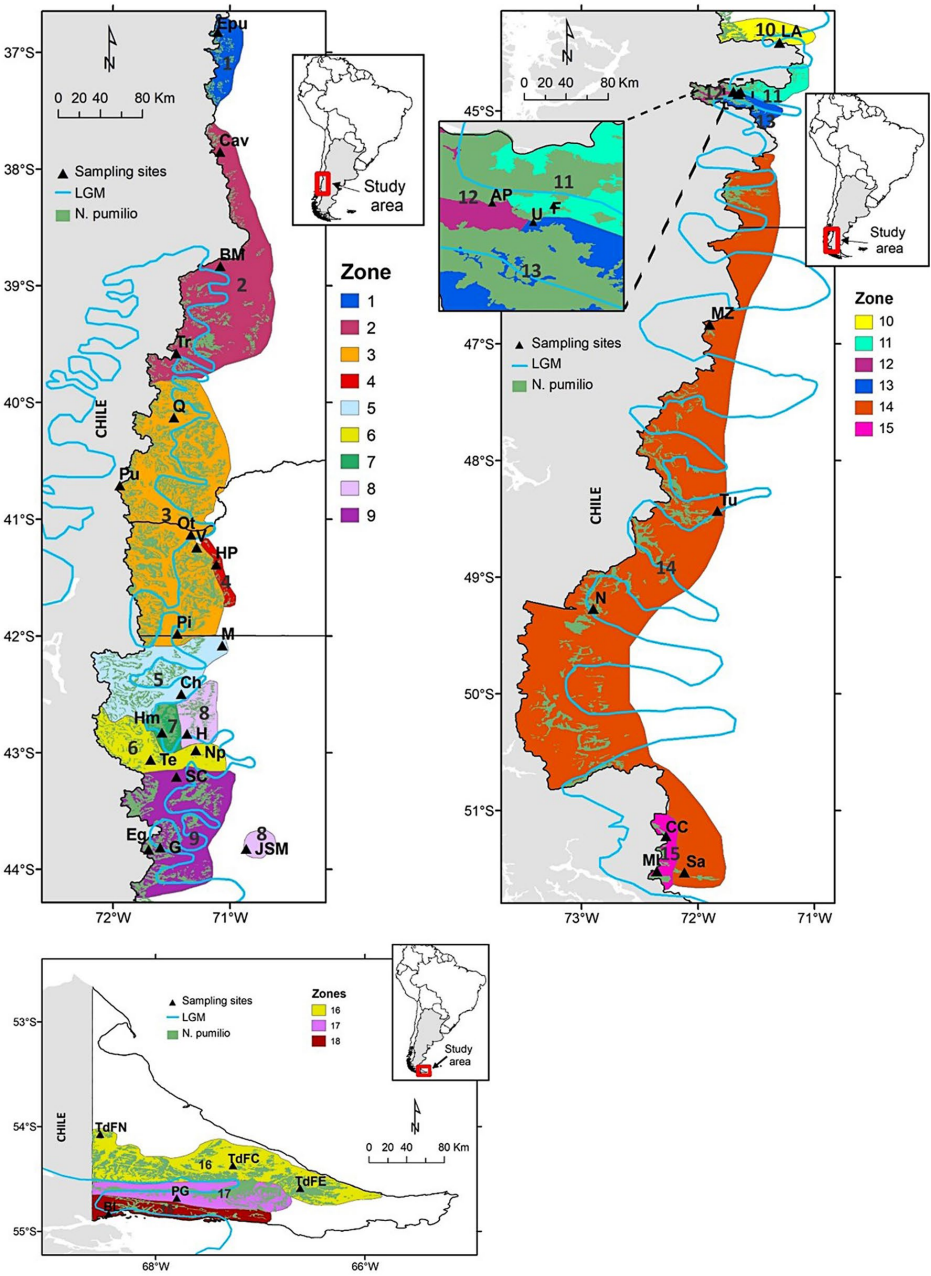
Genetic Zones (GZs) for *Nothofagus pumilio* based on genetic criteria. LGM line corresponds to the Last Glacial Maximum advance (based on Glasser et al.^49^). Maps were created using ArcGIS 9.2 (ESRI, https://www.esri.com).

From the genetic structure analysis (BAPS) of the ‘north’ subset, four main clusters were identified which grouped the populations with an adjustment probability of 0.941 (Supplementary Fig. S2 Ia). In addition, the admixture patterns exhibited in each cluster were taken into account in defining the number of genetic zones that fit best with the structure of *N. pumilio* northern populations (Supplementary Fig. S2 Ib and Ic). Based on this, an extra division was considered within the largest cluster that extends between 37° 51*′*S and 41° 58*′*S. In agreement with these results, five genetic zones were defined which grouped the populations as follows: **GZ1** Epu; **GZ2** Cav, BM, and Tr; **GZ3** Q, Pu, Ot, V, and Pi; **GZ4** HP; and **GZ5** M and Ch (Fig. 3). At the time the genetic diversity for these GZs was studied, it was found that GZ2 and GZ3 presented high *A*_*R*_, *LCA*, and *P*_*a*_ (Table 2). In this regard, several studies suggested that the glacial setting in northern Patagonia led to a higher occurrence of potential refuges at these latitudes than in the southern area^42,43^, as well as more favorable slope-down habitats^44^. The Lácar Lake region (included in GZ3) was reported as a putative relictual zone^33^ since it exhibits high genetic diversity in plants (e.g., *Calceolaria polyrhiza*^45^) and animals (e.g., lizards^46^).

**Table 2:**
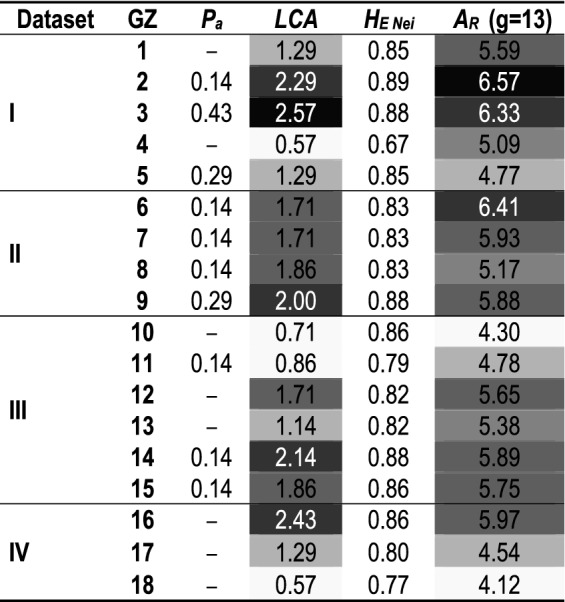
Genetic characterization of the genetic zones.

*GZ* genetic zone, *P*_*a*_ private alleles, *LCA* locally common alleles, *H*_*E Nei*_ Nei’s unbiased heterozygosity obtained through bootstrap re-sampling procedure, *A*_*R*_ allelic richness with g=rarefaction number. I: Target area between 36°S and 42° 30*′*S, II: Target area from 42°50*′*S to 44°S, III: Target area between 44°S and 52°S, and IV: Target area belonging to Tierra del Fuego Island. Gray scale (from black to white) was used for better visualization of the decreasing order in *LCA* and *A*_*R*_ values.

Since the ‘south’ subset comprises a large area with a diverse evolutionary history (i. e., the presence or absence of glacial refugia and post-glacial recolonization patterns), other division criteria were also considered in defining the optimum number of clusters. A particular topographic configuration occurs at 44°S where there are changes in the orientation of the watersheds (north–south oriented mountain chains between 42°S and 44°S shift afterward to a west–east orientation). This feature contributed to different ice mass development during the glacial period^47^, which led to a particular arrangement of ecological niches and refugia^48^. Because of these peculiarities, the populations located between 42° 30*′*S and 44°S (seven populations) were analyzed separately (Fig. 2). The genetic structure analysis of these populations allows us to identify the three main clusters containing them (K = 3 with a probability of 0.865) (Supplementary Fig. S2 IIa). In addition, the admixture test suggested a greater degree of division by identifying a particular situation in Huemules (Hm) (located in the ‘Cordón Rivadavia’ area) which exhibits an almost balanced admixture of K1 and K2 clusters (Supplementary Fig. S2 IIb and IIc). In this way, four GZs were delineated for the area between 42° 30*′*S and 44°S: **GZ6** Te and Np; **GZ7** Hm; **GZ8** H and JSM; and **GZ9** SC, G, and Eg (Fig. 3). GZ6 (including Np and Te populations) showed high genetic diversity (Table 2), which could be explained, at least in part, by the occurrence of hybridization between *N. pumilio* and *N. antarctica* sympatric populations, as reported by Soliani et al.^19^. In addition, high *LCA* and *P*_*a*_ were found in GZ9 (Table 2).

The glacial history south of 44°S showed different processes than the north, leading to the development of a greater ice mass at LGM in this area^2, 4, 49^. In addition, palynological records suggest a different paleoclimatic history for the area south of 50°S, which shaped the forest expansion in a different way (and could also have imprinted the genetic patterns) in southern Patagonia and Tierra del Fuego during the postglacial period^3^. Furthermore, a particular recolonization process was suggested for Tierra del Fuego Island, mainly led by trees from local periglacial refugia^50^. Currently, the *N. pumilio* Argentinean forest south of 44°S is highly fragmented along a narrow range on the Andes hillsides. Nonetheless, an exception to this configuration occurs on Tierra del Fuego Island, where *N. pumilio* forest probably retained or recovered its continuity. Moreover, this forest mass is present at low altitudes (less than 600 m a. s. l.) and is highly influenced by seasonal storm track patterns. In this study the five populations located in Tierra del Fuego were analyzed separately (Fig. 2), considering all the information previously mentioned.

*N. pumilio* populations located south of 44°S, excluding those from Tierra del Fuego, were grouped in four clusters by BAPS (Supplementary Fig. S2 IIIa). In addition, admixture among gene pools was found in the ‘Alto Río Senguer’ basin (around 44° 50*′*S) (Supplementary S2. S2 IIIb and IIIc). In particular, the Union (U) population displayed genetic mixing between Arroyo Perdido (AP) and Fontana (F) gene pools. Based on this, six genetic zones were delineated between 44°S and 52°S: **GZ10** LA; **GZ11** F; **GZ12** AP; **GZ13** U; **GZ14** MZ, Tu, N, and Sa; and **GZ15** CC and MI (Fig. 3). Within this large area, a high *LCA* (2.14) was found only in GZ14 (Table 2), although allelic richness was at least similar in all GZs and even smaller than those obtained for northern GZs.

Populations located on Tierra del Fuego Island were structured in three main clusters (Supplementary Fig. S2 IVa) which also exhibited three distinctive genetic patterns in the STRUCTURE analysis (Supplementary Fig. S2 IVb and IVc). Therefore, three genetic zones were delineated: **GZ16** TdFN, TdFC, and TdFE; **GZ17** PG (under provincial jurisdiction), and **GZ18** BL (situated within a national park) (Fig. 3). *N. pumilio* forest located in the northern region of the island, belonging to GZ16, exhibited high *LCA* and an absence of *P*_*a*_ (Table 2). Additionally, this area (GZ16) was categorized by the provincial government as having medium conservation value, and is currently under management by the forestry industry.

The optimum number of genetic management units (in this case GZs) in *N. pumilio* was confirmed by assessing the hierarchical analysis of variance (AMOVA), where the proportion of variance due to both the ‘among- and within-’ group components were used as elements of comparison. Our proposal of eighteen GZs had a greater among-group than within-group component of variance, which is evidence of correct grouping (Table 3). The phylogenetic relationships among all inferred clusters are presented in Supplementary Figure S3. In addition, some genetic parameters used to characterize these ‘ad hoc*’* GZs (Table 2) are considered in the next section in order to define specific operating practices.

**Table 3:** Analysis of molecular variance (AMOVA) considering 18 Genetic Zones.

Source of variation	SS	VC	%	F-stats	Value	Prob.
Among groups	304.43	0.14	5.71	*F*_*CT*_	0.057	0.000
Among populations w/groups	88.00	0.05	2.20	*F*_*SC*_	0.023	0.000
Among individuals w/populations	2154.54	0.55	23.03	*F*_*IS*_	0.250	0.000
Within individuals	1404.50	1.64	69.05	*F*_*IT*_	0.309	0.000

*SS* sum of squares, *VC* variance components, *%* percentage of the estimated variance, *F*_*CT*_ genetic variability among groups, *F*_*SC*_ genetic variability among populations within groups, *F*_*IS*_ genetic variability within populations, *F*_*IT*_ total genetic variability.

### Priority conservation areas and operational management considerations

The delineated GZs could facilitate the implementation of specific practices related to conservation programs by determining priority areas based on the genetic diversity of each OGMU. The genetic structure of *N. pumilio* was assessed here by neutral molecular markers (SSRs) and was taken into account when delineating *N. pumilio* GZs in Argentina. This approach has been successfully used to define management units for several species (e.g.,^25,51,52^), since microsatellite diversity was correlated with genomic diversity at population level, although not at individual level^53^. In addition, considering that microsatellite diversity may not reflect adaptive diversity^54^, when defining Conservation Units (CUs) and Provenance Regions (PRs) it would be appropriate to determine the genetic variation data based on adaptive traits^30,55^. Even though Chilean *N. pumilio* populations were not sampled, the current status of these forests could reflect similar variation to the Argentinean side, since they are probably subject to comparable threats. However, it may be difficult to achieve common management criteria on both sides of the mountain range (Andes Cordillera), since each country has specific regulations.

Standardized allelic richness (*A*_*R*_) is commonly chosen as a genetic criterion in order to define priority areas for conservation^31,32^. This parameter is independent of sample size^31,56^ and suitable for determining historical processes such as bottleneck or admixture^56^, and inferring putative relictual zones^31^. Accordingly, GZ2, GZ3, and GZ6 require special attention due to their high genetic diversity (high standardized *A*_*R*_, Table 2). Therefore, these areas become meaningful for in situ and ex situ conservation purposes, especially since they play a key role as genetic diversity reservoirs. In this sense, a high number of genetic variants with great potential for adapting to climate change could be present in these reservoir areas^57,58^. They are therefore essential as a source of planting material, to ensure the long-term success of active restoration programs^59,60^ and to enrich low-intensity breeding programs.

Numerous studies suggest that priority should also be given to areas exhibiting locally common or exclusive alleles (i.e. *P*_*a*_), which could be evidence of genotypes adapted to specific environments^30,60^. Furthermore, a high *LCA* value could be indicative of genetic isolation^30^ and thus help to identify the putative location of species–specific refugia^61,62^. Based on the high *LCA* and/or *P*_*a*_ detected in this study (Table 2), **GZ2**, **GZ3**, **GZ9**, **GZ14**, and **GZ16** should be highlighted as holding exclusive allelic variants. In this regard, in situ conservation management is highly encouraged for these genetic zones. Furthermore, tree-planting activities targeting these areas should preferably use reproductive material collected from local sources.

In addition, since a relatively recent demographic bottleneck was inferred in the *N. pumilio* forest located close to Rosario Lake (SC population), particular consideration should be given to this area, which is included within GZ9. This demographic event leads to a reduction in genetic diversity, which could threaten the adaptive potential of this population and, in an extreme case, lead to local extinction^63^. For this reason, strict in situ conservation management is highly recommended for this area, in order to prevent the loss of these genetic variants.

Finally, GZ7 and GZ13 displayed specific genetic composition, based on their genetic structure and admixture patterns. In particular, these zones combine an almost balanced mixture of two surrounding gene pools, and thus, special consideration is suggested for their operational forest management. Local sources must be chosen if reproductive materials are required for active restoration in these GZs, in order to preserve their characteristic genetic structures. In the case of severe loss of the GZ7 and GZ13 local forests (e.g., caused by an intensive fire), a balanced seed pool from the surrounding GZs is suggested for future tree-planting activities.

### Final remarks

This study presents the first scientific evidence for the definition of a *N. pumilio* management plan based on genetic knowledge and considering its entire distribution range (more than 2200 km). Six priority areas (GZ2, GZ3, GZ6, GZ9, GZ14 and GZ16) were identified that could lead conservation action, specifically targeting in situ conservation. Furthermore, this study enabled the delineation of 18 *N. pumilio* GZs in Argentina, thus providing preliminary operational genetic management units that best fit the genetic structure of the target species. Maps showing the distributional range of each GZ in Argentina were drawn up. Reproductive material can thus be transferred within these ad hoc GZs with reduced risk of altering the original genetic structure. This possibility is important in light of the numerous active ecological restoration projects that have been developing in the *N. pumilio* forests on both sides of the Andes Range in the last decade. In addition, the recommendations suggested here are highly important for regulatory institutions that have to enforce compliance with national Law 26331, on the protection of native forests. Moreover, the 18 GZs constitute a first step towards the definition of both PRs and CUs, thus promoting preservation of the southernmost forest ecosystem of the world.

## Materials and methods

### Plant material and molecular analysis

Of the 35 *N. pumilio* populations (965 adult trees) included in this study (Table 1, Fig. 1), 20 had been previously analyzed to determine the species’ phylogeographic patterns^19,33^, while the remaining 15 populations were used exclusively in the present work. The sampled sites covered the natural distribution area of the target species in Argentina from 36°S to 55°S in the latitudinal range (more than 2200 km) and from 71° 56′ to 66° 37*′* in the longitudinal range. A minimum of 20 trees were sampled in each population, with the exception of Cholila and El Chalten sites where 18 and 19 samples were obtained (respectively) due to limited access. In every site, a minimum distance of 50 m between sampled trees was maintained in order to preclude the collection of closely-related individuals. In addition, only *N. pumilio* phenotypes were considered at the time of population sampling (thus, avoiding possible or ambiguous intermediate phenotypes between this species and related ones).

Total DNA was extracted from bud tissue following the procedure described by Dumolin et al.^64^. Samples were stored at − 20 °C until PCR amplifications were carried out. Seven polymorphic loci (Npum1, Npum3, Npum9, Npum10, Npum13, Npum17a, and Npum18) were analyzed using a specific set of microsatellite markers (nuSSRs) developed for this species by Soliani et al.^65^. SSR amplification conditions and PCR thermal profiles were set following the recommendations given by the authors^65^. Amplification reactions were performed with 20 ng of template DNA in a final volume of 11 µl, using 1X Green Buffer and GoTaq DNA Polymerase (Promega, Wisconsin, USA) for all primers, with the exception of Npum10 that was amplified with Platinum Taq (Invitrogen, California, USA). The M13 protocol^66^ was applied and PCR products were visualized on 1% agarose gel stained with Sybr Safe (Invitrogen). Individual genotyping was done using a 3130xl Genetic Analyzer sequencer (Applied Biosystems, California, USA) and the allele assignment with GeneMarker V2.7.4 (SoftGenetics, demo version).

### Genetic characterization of the populations

A set of genetic parameters was first obtained in order to characterize the populations under study (Table 1). These parameters included the number of different alleles (*N*_*a*_), the effective number of alleles (*N*_*e*_), and the expected heterozygosity that considers the null allele effect (*H*_*E null*_), which were obtained with GenAlEx 6.5 software^67^. Unbiased heterozygosity described by Nei^68^ was also estimated with Info-Gen software^69^. The Wilcoxon test was implemented as an a posteriori multiple-comparison analysis, to identify the significant differences among populations. The presence of null alleles at both locus and population levels was tested with MICRO-CHECKER^70^ and their frequencies were estimated using FreeNA^71^. The presence of null alleles was considered in the estimation of inbreeding coefficients (*F*_*IS*_), which was calculated with INEST 2.2^72^. Two approaches were tested for obtaining *F*_*IS*_: 1. an Individual Inbreeding Model (IIM) using a Bayesian approach (*F*_*IS*_*′*), and 2. a Maximum Likelihood approach (*F*_*IS*_*”*). In both cases, three different models were tested (i. e., considering the occurrence of null alleles, genotyping errors, and inbreeding events) at population level, and the model with the lowest Deviance Information Criterion (DIC) was considered as best fitting the data. Once the inbreeding model had been selected, the mean inbreeding coefficients (*F*_*IS*_*′)* were calculated. In addition, a jackknife procedure was performed to estimate the standard error (SE) of the *F*_*IS*_*”* values, which were then used to assess the significance of this coefficient using the Z-test based on the normal distribution theory.

In order to identify populations experiencing a relatively recent reduction in effective population size, a demographic bottleneck test was carried out using BOTTLENECK software^73^. This approach computes the distribution of the expected heterozygosity from the observed number of alleles at population level, assuming the mutation-drift equilibrium model. A significant heterozygosity excess or deficiency is confirmed by performing a Wilcoxon sign-rank test for every mutation model (i.e., Infinite Allele Model -IAM, Stepwise Mutation Model -SMM, and Two Phase Model -TPM). In this study, a past population bottleneck was inferred when heterozygosity excess was significant (*p* < 0.05) in at least two of the above-mentioned mutational models.

Genetic differentiation among populations was determined by estimating both *F*_*ST*_^74^ and *G′*_*ST*_^75^ indexes. *F*_*ST*_ was then recalculated by implementing the ‘exclusion of null alleles’ (ENA) method in order to avoid a possible bias introduced by the presence of null alleles^71^. Confidence intervals (95% level) were obtained through a bootstrap re-sampling procedure. In addition, to evaluate the occurrence of an isolation-by-distance (IBD) pattern, a correlation between the genetic and geographic distances between populations was assessed using two approaches: a. The Mantel test, performed with Info-Gen and using the correlation between *F*_*ST*_ estimates and the logarithm of the geographic distances between population pairs; b. The Spatial Genetic Structure (SGC) test by GenAlEx 6.4, with which spatial autocorrelation coefficients (***r***^76^,) were obtained for ten distance classes (each one covering 200 km). In both approaches, 1000 permutations were considered when calculating statistical significance. In the SGC test, the upper and lower error limits for the 95% confidence interval were determined by carrying out 10,000 bootstrap samples.

### Population genetic structure and cluster definition

Due to the large latitudinal span of the area under study (more than 2200 km long) and the diverse evolutionary history of the region, multiple division criteria were considered (Fig. 2). Firstly, previous information from chloroplast DNA (cpDNA) markers(^33^, see Fig. 2a) was used, and the haplotypes of new samples from the Cholila population (~ 42°S) were determined due to its proximity to a transition zone between the northern and southern lineages. In a second stage, a broad-scale clustering analysis was performed (considering the 35 populations) using a Bayesian approach by implementing BAPS 6.0 software^77^. This analysis showed weak grouping, since the adjusted probability of the inferred number of clusters was 0.58632. This reinforced the decision of considering alternative division criteria before performing the final clustering.

A phylogeographic break around 42°S latitude was previously reported for some species of *Nothofagus* subgenus *Nothofagus*, which was inferred from genetic studies using cpDNA markers^19,33,39,78^. It was suggested that northern and southern lineages were geographically segregated around 42°S and were probably isolated for a prolonged period, which indicates a different evolutionary history. In addition, different glaciation patterns were described for the areas located between 38°–42°S and areas southward of 45°S^49^. A particular distribution of *N. pumilio* forests is currently characteristic from 41°S northward, since it occurs as a continuous forest mass in the subalpine zone, forming the upper tree limits, whereas at the southern latitudes it also occurs at lower elevations^11^. Based on all this information, the studied populations were divided into two subsets for further analyses: 1. ‘North’ including the 12 populations with northern cpDNA haplotypes, located between Epulauquen (36° 49*′*S) and Cholila, and 2. ‘South’ including the remaining 23 populations that exhibited southern cpDNA haplotypes and are located between Huemules and Bahía Lapataia (54° 50*′*S). The genetic structure of *N. pumilio* populations from each north and south group was then determined by a spatial clustering method using BAPS software. This approach has the advantage of considering the geographic locations of pre-assigned groups of individuals when defining the best number of clusters (K)^77^. In addition to this analysis, an admixture model within each inferred K was tested with STRUCTURE 2.2 software^79^ by running 500,000 Markov chain Monte Carlo (MCMC) steps, a burn-in of 100,000 iterations and five replicates for each inferred K in the entire analysis. Complementarily, UPGMA (Unweighted Pair Group Method using Arithmetic averages) dendrograms based on Nei’s distance (averaged over loci) were constructed to explore the relationships among the identified clusters.

A grid-based spatial analysis was carried out with a resolution of 2 min to visualize the geographical distribution and admixture pattern of inferred clusters. Separate raster maps were produced, considering a circular neighborhood cell of 20 min diameter around every sampled location, following van Zonneveld et al.^32^ and Thomas et al.^80^. In this case, individual admixture coefficients for each inferred cluster (obtained by STRUCTURE) larger than 0.7 were considered. Each cell was assigned with the mean membership value of the individuals, enclosed by a circular neighborhood of 20 min diameter constructed around its center to graphically represent the inferred clusters. Raster package 2.14 was used to generate these maps in R software environment (R Core Team) and they were edited in ArcGIS 9.3 (ESRI, Redlands, California, USA).

### Delineation and characterization of the genetic zones

Delineation of GZs was performed based on the previously-mentioned analyses and summarized in Fig. 2. Information on historical processes such as the putative location of glacial refugia and post-glacial recolonization patterns was taken into account in identifying the optimum number of clusters. In addition, other criteria such as topography, mountain height and the presence of water bodies (e.g., lakes) were considered.

A hierarchical analysis of molecular variance (AMOVA) was performed using Arlequin 3.5^81^, in order to evaluate the number of clusters that best grouped the studied populations. The optimum number of genetic zones was confirmed based on the significant differences among and within the obtained clusters, as proposed by Pastorino and Gallo^20^; i.e., genetic variance should be maximum between populations of different groups and minimum within the groups.

To obtain valuable information for the definition of management plans, some genetic parameters for each defined GZ were calculated with GenAlEx and Info-Gen: private alleles (*P*_*a*_), the expected heterozygosity considering the null allele effect (*H*_*E null*_), and locally common alleles (*LCA*). The *LCA* average for each GZ was calculated considering alleles with frequencies higher than 5% and, at the same time, an occurrence probability of less than 25% of the defined GZs. In addition, genetic diversity was estimated by calculating allelic richness (*A*_*R*_) after rarefaction to a common sample size^82^ using FSTAT software^83^.

## Supplementary information

Supplementary Information 1.

Supplementary Information 2.

Supplementary Information 3.
