# Supplementary material for: Genetic diversity and population structure in Nothofagus pumilio, a foundation species of Patagonian forests: defining priority conservation areas and management
Source: Sci Rep. 2020 Nov 6;10:19231. doi: 10.1038/s41598-020-76096-0 (PMC7648826; doi:10.1038/s41598-020-76096-0)

**Genetic diversity and population structure in *Nothofagus pumilio*, a foundation species of Patagonian Forests: defining priority conservation areas and management**

Ma. Gabriela Mattera[1*], Mario J. Pastorino[1], Ma. Victoria Lantschner[2], Paula Marchelli[1], and Carolina Soliani[1]

[1]Grupo de Genética Ecológica y Mejoramiento Forestal del Instituto de Investigaciones Forestales y Agropecuarias Bariloche (IFAB) INTA EEA Bariloche –CONICET, [2]Grupo de Ecología de Poblaciones de Insectos del Instituto de Investigaciones Forestales y Agropecuarias Bariloche (IFAB) INTA EEA Bariloche –CONICET

*mattera.gabriela@inta.gob.ar; Modesta Victoria 4450, CP8400, S. C. de Bariloche, Río Negro, Argentina.

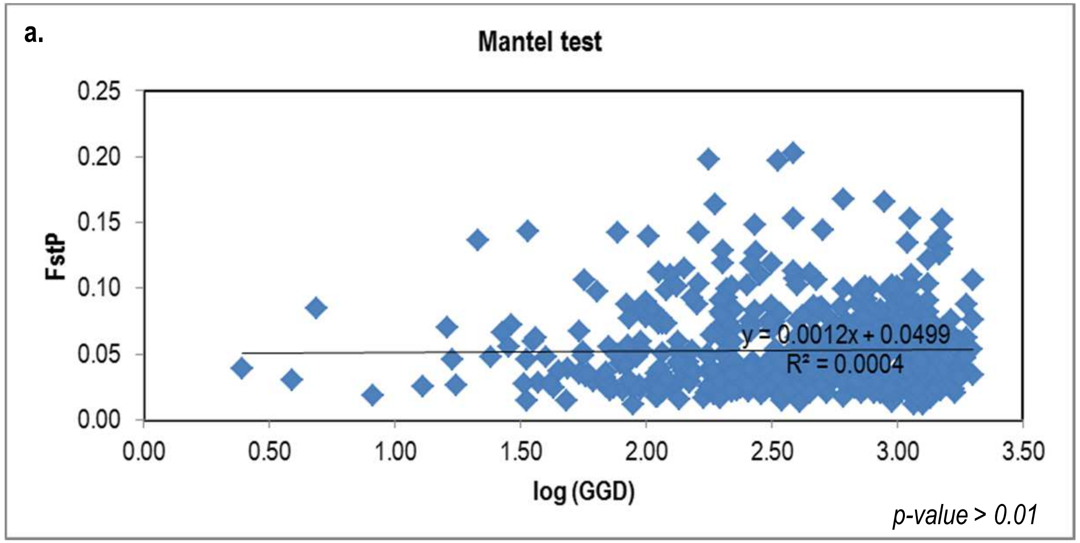


**Supplementary Fig. S1**. Isolation by distance (IBD) analysis. **a.** Mantel Test, **FstP**= differentiation coefficients between population pairs, **log (GGD)**= logarithm of geographical distances between population pairs; **b.** Spatial Genetic Structure test, ***r***= autocorrelation coefficient, **U**= upper limit for the 95% confidence interval, **L**= lower limit for the 95% confidence interval.

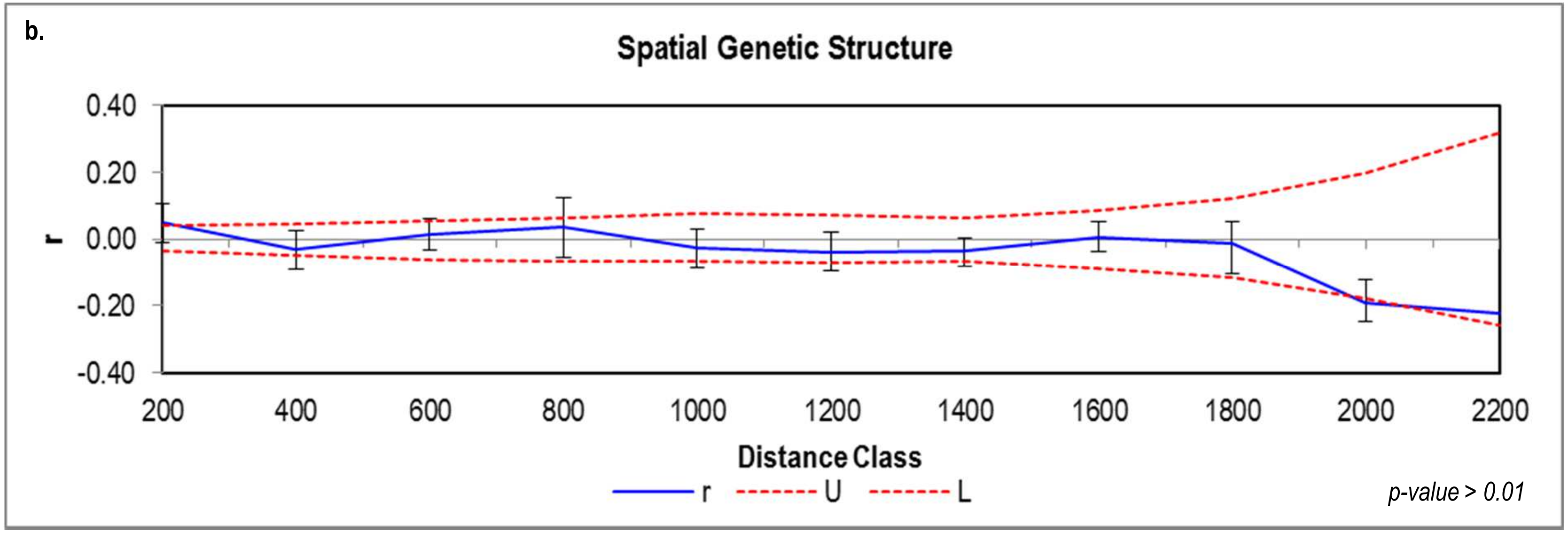

Supplement: Supplementary file 1 — Supplementary Information 1. [file 41598_2020_76096_MOESM1_ESM.pdf]
