# Supplementary material for: Genetic diversity and population structure in Nothofagus pumilio, a foundation species of Patagonian forests: defining priority conservation areas and management
Source: Sci Rep. 2020 Nov 6;10:19231. doi: 10.1038/s41598-020-76096-0 (PMC7648826; doi:10.1038/s41598-020-76096-0)
Supplement: Supplementary file 2 — Supplementary Information 2. [file 41598_2020_76096_MOESM2_ESM.pdf]

Genetic diversity and population structure in *Nothofagus pumilio*, a foundation species of Patagonian Forests: defining priority conservation areas and management

Ma. Gabriela Mattera[1*], Mario J. Pastorino[1], Ma. Victoria Lantschner[2], Paula Marchelli[1], and Carolina Soliani[1]

[1]Grupo de Genética Ecológica y Mejoramiento Forestal del Instituto de Investigaciones Forestales y Agropecuarias Bariloche (IFAB) INTA EEA Bariloche –CONICET, [2]Grupo de Ecología de Poblaciones de Insectos del Instituto de Investigaciones Forestales y Agropecuarias Bariloche (IFAB) INTA EEA Bariloche –CONICET

*mattera.gabriela@inta.gob.ar; Modesta Victoria 4450, CP8400, S. C. de Bariloche, Río Negro, Argentina.

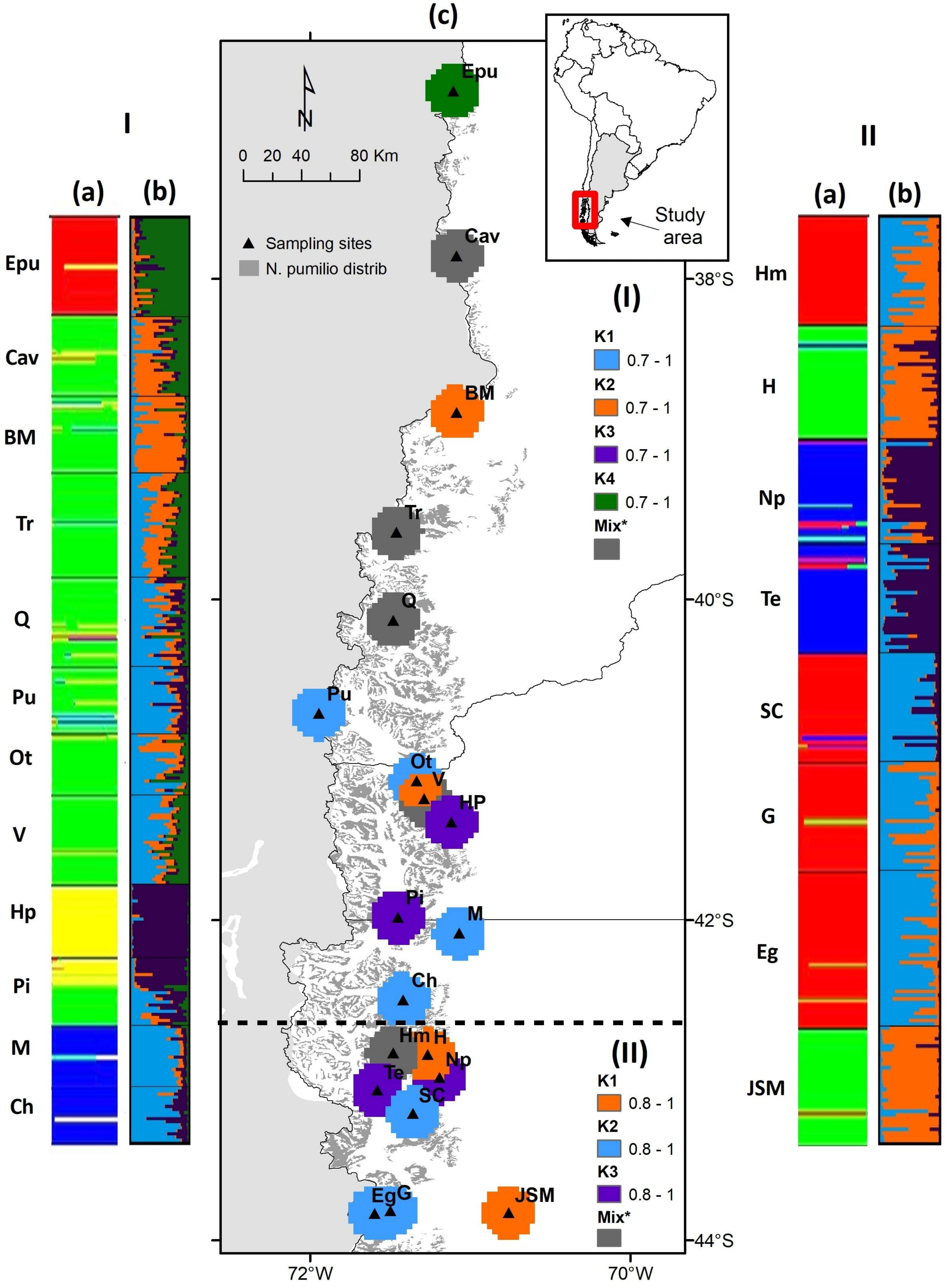

I
(a)
(b)
Epu
Cav
BM
Tr
Q
Pu
Ot
V
Hp
Pi
M
Ch
(c)
N
0 20 40 80 Km
Sampling sites
N. pumilio distrib
Study area
38°S
40°S
42°S
44°S
72°W
70°W
Epu
Cav
BM
Tr
Q
Pu
Ot
V
HP
Pi
M
Ch
Hm
H
Np
Te
SC
Eg
G
JSM
(I)
K1
0.7 - 1
K2
0.7 - 1
K3
0.7 - 1
K4
0.7 - 1
Mix*
(II)
K1
0.8 - 1
K2
0.8 - 1
K3
0.8 - 1
Mix*
II
(a)
(b)
Hm
H
Np
Te
SC
G
Eg
JSM

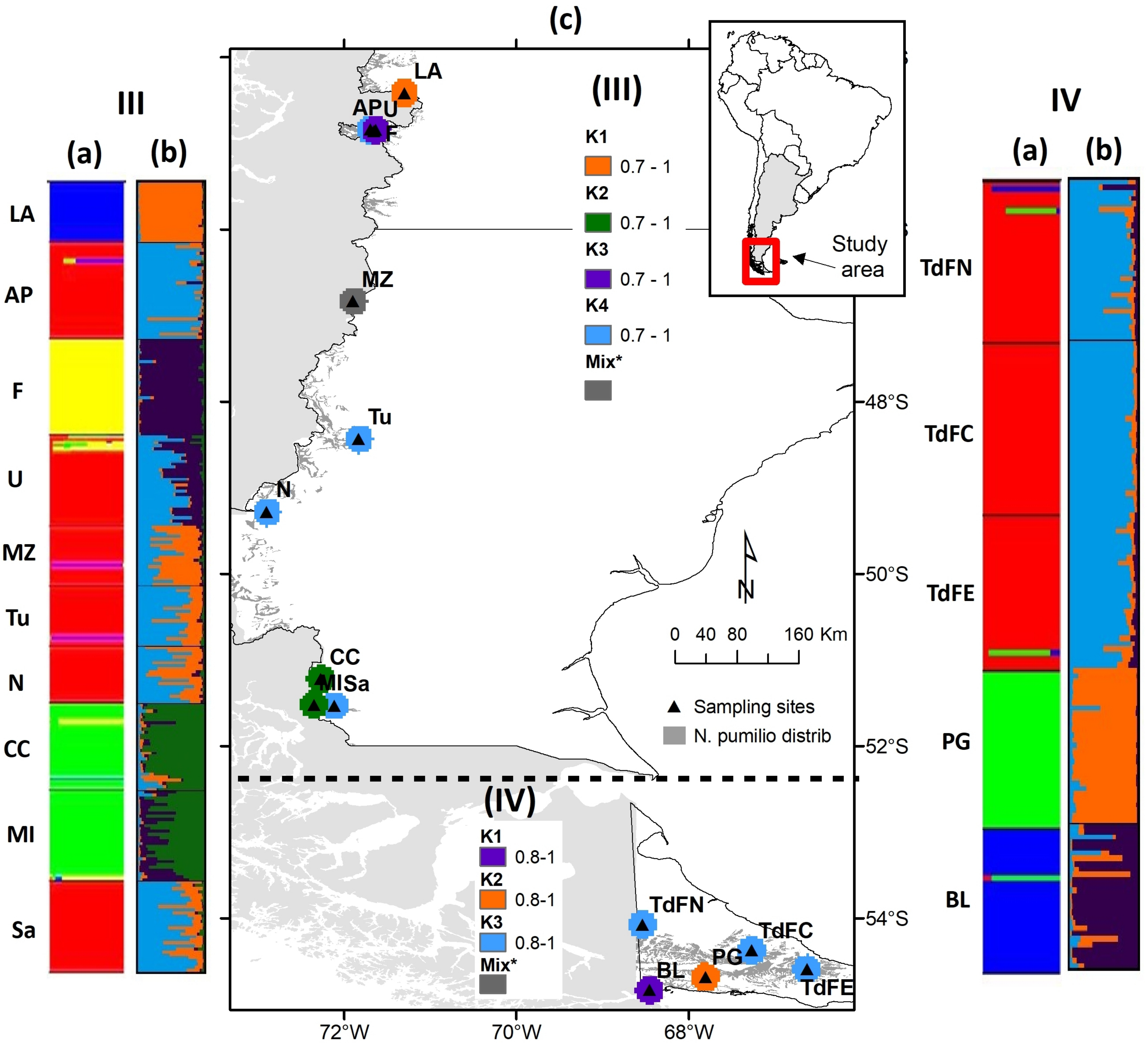

Supplementary Fig. S2. Set of analyses leading to determination of the optimum number of clusters (K) and delineation of N. pumilio GZs. I: target area between 36ºS and 42º 30´S, II: target area from 42º50´S to 44ºS, III: target area between 44ºS and 52ºS, and IV: target area corresponding to Tierra del Fuego Island. 'Mix'* indicates that this area combines two or more clusters. (a) Analysis of the genetic structure of populations using BAPS software, (b) Analysis of admixture patterns using STRUCTURE (tested K range by area: 1 – 4 in areas I, II, and III, but 1 – 3 in area IV), (c) Grid-based spatial analysis using STRUCTURE admixture coefficients for each inferred cluster (colors correspond to those presented in (b)). Raster package 2.14 was used to generate these maps in R software environment (R Core Team) and they were edited in ArcGIS 9.3 (ESRI, https://www.esri.com).
