# Supplementary material for: Genetic diversity and population structure in Nothofagus pumilio, a foundation species of Patagonian forests: defining priority conservation areas and management
Source: Sci Rep. 2020 Nov 6;10:19231. doi: 10.1038/s41598-020-76096-0 (PMC7648826; doi:10.1038/s41598-020-76096-0)
Supplement: Supplementary file 3 — Supplementary Information 3. [file 41598_2020_76096_MOESM3_ESM.pdf]

Genetic diversity and population structure in *Nothofagus pumilio*, a foundation species of Patagonian Forests: defining priority conservation areas and management

Ma. Gabriela Mattera[1*], Mario J. Pastorino[1], Ma. Victoria Lantschner[2], Paula Marchelli[1], and Carolina Soliani[1]

[1]Grupo de Genética Ecológica y Mejoramiento Forestal del Instituto de Investigaciones Forestales y Agropecuarias Bariloche (IFAB) INTA EEA Bariloche –CONICET, [2]Grupo de Ecología de Poblaciones de Insectos del Instituto de Investigaciones Forestales y Agropecuarias Bariloche (IFAB) INTA EEA Bariloche –CONICET

*mattera.gabriela@inta.gob.ar; Modesta Victoria 4450, CP8400, S. C. de Bariloche, Río Negro, Argentina.

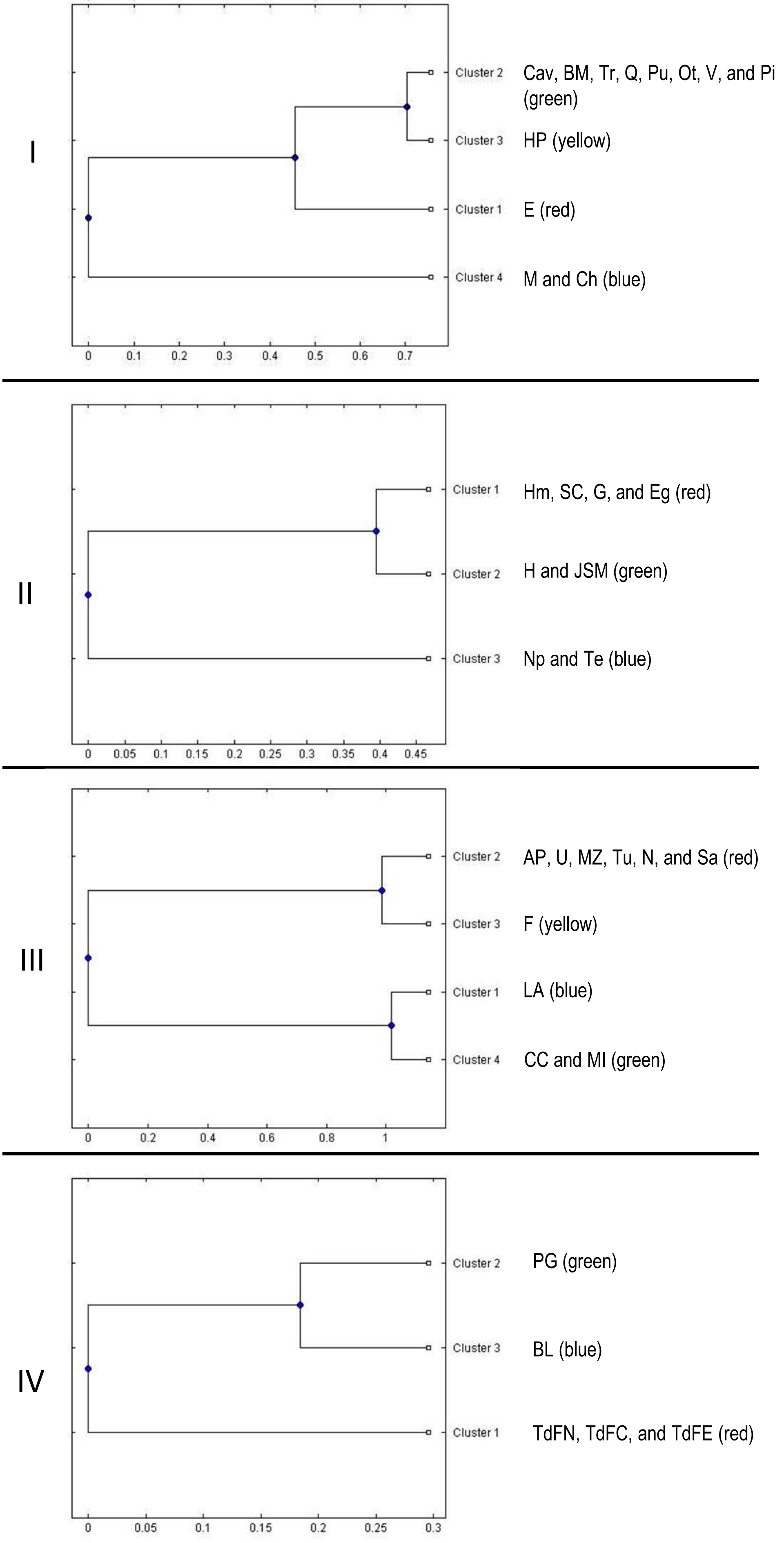

Supplementary Fig. S3. UPGMA dendrograms based on Nei´s distance (average over loci). Every cluster corresponds to those shown in Supplementary Fig. S2 Ia, IIa, IIIa, and IVa. I: target area between 36ºS and 42º 30´S, II: target area from 42º50´S to 44ºS, III: target area between 44ºS and 52ºS, and IV: target area corresponding to Tierra del Fuego Island. These phylogenetic trees allowed us to understand the relationships between the identified clusters.
